# Potential Application of Black Soldier Fly Larva Bins in Treating Food Waste

**DOI:** 10.3390/insects14050434

**Published:** 2023-05-02

**Authors:** Irfana Kabir Ahmad, Ng Teck Peng, Nur Fardilla Amrul, Noor Ezlin Ahmad Basri, Nurul Ain Abdul Jalil, Nur Asyiqin Azman

**Affiliations:** 1 Department of Civil Engineering, Faculty of Engineering and Built Environment, Universiti Kebangsaan Malaysia, Bangi 43600, Selangor, Malaysia; 2 Sustainable Urban Transport Research Centre (SUTRA), Faculty of Engineering and Built Environment, Universiti Kebangsaan Malaysia, Bangi 43600, Selangor, Malaysia; 3 Department of Earth Science and Environment, Faculty of Science and Technology, Universiti Kebangsaan Malaysia, Bangi 43600, Selangor, Malaysia

**Keywords:** *Hemertia illucens*, black soldier fly bin design, chicken feed, composting, organic waste

## Abstract

**Simple Summary:**

As a converter of organic waste, the black soldier fly (BSF), *Hermetia illucens Linnaeus* (Diptera: Stratiomyidae), has been introduced, especially to deal with the increasing organic waste generation and the number of landfills. In this study, we designed a user-friendly BSF larvae (BSFL) bin that the public and institutions can fabricate and use as an effort to reduce waste. We also tested mixtures of food waste and moisture content control medium (MCCM) as larval feeding mediums to ease the handling and maintenance of the bins and identify the optimum medium for larval growth. Our findings show that the fabricated BSFL bins can fulfil the BSF lifecycle requirements. The medium without MCCM produced the highest larval growth; however, the high moisture content of the medium causes difficulty in handling and managing the bins. Overall, the mediums with MCCM produced lower moisture, which makes bins easier to maintain, and it is proven in this study that chicken feed is the most suitable MCCM as it produces the optimum larval growth and frass moisture.

**Abstract:**

The increase in the global population has led to a rise in organic waste generation and landfill sites. Consequently, there has been a global shift in focus towards the utilization of BSFL to address these challenges. This study aims to design, develop, and test a user-friendly BSFL bin and identify the optimal MCCM for treating organic waste using BSFL. The four BSFL bins have a dimension of 330 mm (width) × 440 mm (length) × 285 mm (height). This study uses mixtures of food waste added with different MCCMs, including chicken feed, rice bran, and garden waste. We add the mediums to the BSFL bins every third day and measure the humidity, ambient temperature, pH, medium temperature, and BSFL weight and length. The measurements show that the fabricated BSFL bins can fulfill the BSF lifecycle requirements. Wild BSFs lay eggs in the medium of BSFL bins, and the hatched larvae decompose it. When they reach the prepupae stage, they climb the ramp into the harvesting container. The results show that the food waste without MCCM produced the heaviest (0.228 g) and longest (2.16 cm) larvae; the prepupae are 2.15 cm long and weigh 0.225 g; and the growth rate is 53.72%. However, the high moisture content of 75.3% makes the maintenance work challenging. The medium with MCCM has a markedly lower moisture content of 51–58%. A comparison of the three MCCMs shows that the chicken feed produces the larvae and prepupae with the highest growth rate (the larvae are 2.10 cm long and weigh 0.224 g, the prepupae are 2.11 cm long and weigh 0.221 g, and the growth rate is 72.36%) and the frass with the lowest moisture content (51.2%). An effective BSFL composting system is easy to manage and produces the biggest larvae. In summary, food waste mixed with chicken feed is the most suitable MCCM for treating organic waste using BSFL.

## 1. Introduction

According to the United Nations [1], the global population is projected to reach 8 billion on 15 November 2022 and could reach 8.5 billion in 2030, 9.7 billion in 2050, and 10.4 billion in 2100. The surge in solid waste generation due to the increase in human population is a grave concern. The current global solid waste generation is approximately 3.9 million tons a day; without a proper solid waste management plan, the volume is projected to increase by 70% in 2050. A survey showed that high-income countries produced higher amounts of food waste than middle- and low-income countries [2]. Developing countries and fast-growing cities will be facing a challenge in food waste management, and Malaysia is one of the developing nations currently struggling with waste management.

According to the Department of Statistics, Malaysia had a population of 32.63 million in 2019, an increase of 38.9% from 23.49 million in 2000 [3]. The Department of Solid Waste Management stated that 45% of the solid waste produced in Malaysia is food waste, and the remaining waste consists of glass, metals, papers, plastics, rubbers, and wood. In 2014, Malaysia generated 8000 tons of food waste daily [4], and the volume increased by 760% from the 930 tons of waste generated in 2011 [5]. Malaysia produced 8000 tons of food waste daily when its population was 30 million. The former Prime Minister Tun Dr Mahathir Mohamad stated that Malaysia must have a population of 70 million by 2050 to be a self-sustaining market [6]. Based on this projection, the volume of food waste generated in 2050 will be at least a few folds higher compared to that of 2014.

Asian countries dispose of their food waste in landfills or burn it manually or in incinerators. These food waste disposal methods are preferred in South East Asia because of financial, social, leachate, and gas emission considerations. Malaysia uses these methods because they have the lowest set-up and maintenance costs, and most landfills are open dumping sites [7]. Landfills are known to cause problems such as pollution of underground water by leachate, land pollution, air pollution from open burning, unpleasant odors affecting the health of the surrounding population, and transmission of pest-borne diseases [8]. The other waste disposal methods adopted by Malaysia, such as recycling and composting, are insignificant because of their low adoption rate [9]. 

In the waste disposal hierarchy, prevention and reduction are at the highest level and are the most preferred methods, while disposal is the least preferred method. There are alternative methods for reducing the waste disposed of in landfills, such as composting using black soldier fly larvae (BSFL). BSFL are more effective than traditional composting in reducing 50% of organic waste within a shorter period since they can consume large amounts of organic waste and decompose plant matter, manure, food waste, and municipal trash for growth until they reach the prepupae stage [9,10,11]. The benefits of composting with BSFL include a reduced amount of organic waste disposed of in the landfills and using the larvae and the prepupae as animal feed for domestic animals, such as chickens, fish, and pigs, since BSFL are rich in protein and nutrients. Several studies on the generation of alternative feed, especially for aquaculture, have been conducted to determine the effects of the use of BSFL in diets and showed that BSF is a promising protein source for aquafeeds [12,13]. A bibliometric analysis by Mangindaan et al. [14] characterized the development of publications over the last 10 years (2011-2022) in order to determine the future research directions of BSFL for animal feed and biodiesel as a solution for the Waste–Food–Energy Nexus, showing the interest of research in this field. In addition, BSFL are also known to increase the phosphorus, nitrogen, and potassium contents in composts, making them suitable plant and vegetable fertilizers [15]. As an example, with the amendment of the substrate with sawdust to a C/N ratio of 15, Beesigamukama et al. [16] demonstrated that recycling agro-industrial waste using BSF could generate nutrient-rich organic fertilizer for organic farming.

This study aims to ensure that this idea becomes a reality in the effort to reduce the food waste disposed of in landfills by designing an appropriate BSFL composting bin that the public and institutions can fabricate and use. This study adds different moisture content control mediums (MCCM) to the food waste to ease the handling and maintenance of the composting bins because, hypothetically, MCCM helps to reduce the moisture of the medium and produce dry residue which has a less foul smell. Despite the increase in the cost of operation by adding MCCMs, it is necessary for providing additional nutrients to the larvae. Thus, this study will also compare the effects of the MCCMs on BSFL growth by measuring the weight and length of the BSFL. The study result will be the basis for selecting the best MCCM for organic waste treatment using BSFL. This study adopts the framework which designs the BSFL bin, prepares the medium, hatches the black soldier fly eggs, treats the food waste, and pre-tests and analyses the data. The parameters for the data analysis are the ambient temperature and humidity; the pH, temperature, moisture content, and waste reduction index of the medium; the weight and length of the larvae and prepupae (weight and length); and other observations. This study hopes to design easy-to-fabricate BSFL composting bins with a simple BSFL composting system handling to create awareness and encourage the public to practice composting organic waste using BSFL.

## 2. Materials and Methods

### 2.1. Design of the Black Solider Fly Larvae Bin

**Figure 1:**
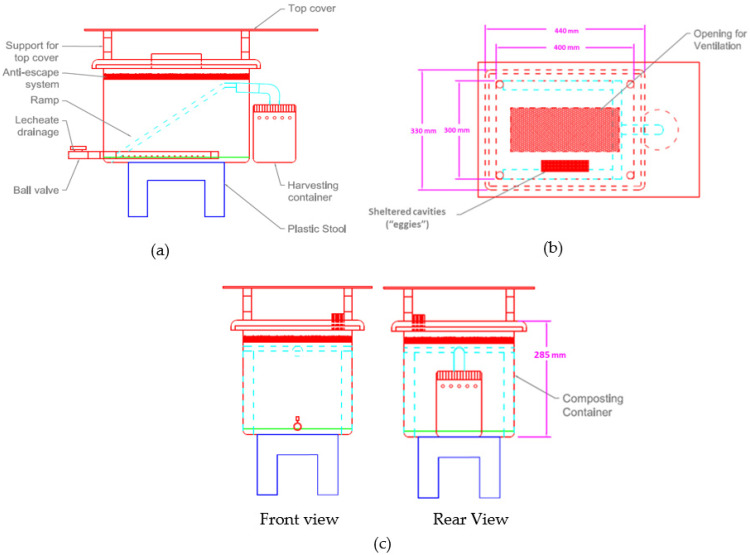
Design of the BSFL bin, (**a**) side view; (**b**) top view; and (**c**) front and rear view.

The design of the BSFL bin in this study was inspired by the currently available bio-pods in the market, published journals, and websites. The materials used to fabricate the BSFL bins are typical household items available in the market to ensure that the public can build the BSFL bins. The dimensions of the BSFL bin are based on the average daily production of organic waste by a typical household to ensure that the frass produced by the larvae does not exceed 10 cm at the end of each cycle. Layers exceeding 10 cm will become anaerobic and unsuitable for the larvae [11]. The Malaysia Ministry of Housing and Local Government reported that urban households produce about 0.37 kg/capita/day of organic waste, while rural households produce about 0.27 kg/capita/day [17]. The calculation showed that a 25 L plastic storage box with a 30 cm × 40 cm (1200 cm^2^) base is perfect for the waste load. The BSFL bin was designed to hold up to 1440 larvae based on the ideal larvae density of 1.2 larva/cm^2^ in the research conducted by Parra Paz et al. [18]. This study fabricated four BSFL bins labelled A, B, C, and D, run for two rearing cycles. Figure 1 presents the bin components.

**Figure 2:**
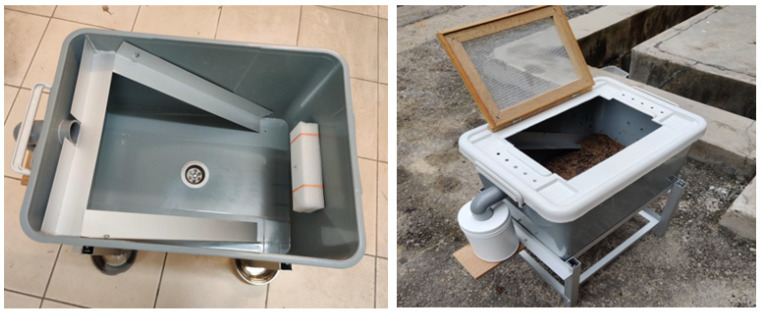
BSFL bin prototype.

Before carrying out the actual experiment, we ran a pre-test using the prototype BSFL bin and finalized its design. The pre-test used about 2000 larvae, and we added 1 kg of food waste daily for two weeks. The pre-test ensured that the design and materials for the prototype bin were suitable for the experiment. Figure 2 shows the prototype BSFL bin.

**Figure 3:**
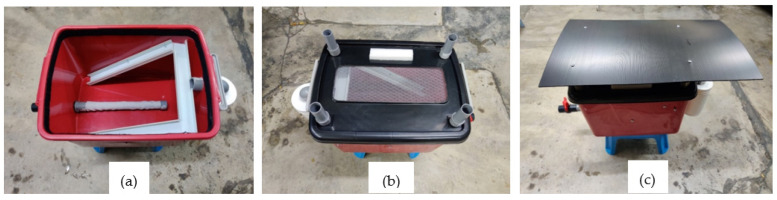
The fabricated BSFL bin, (**a**) inside bin; (**b**) lid without roof; and (**c**) lid with roof.

Several problems were identified during the two weeks of the pre-test and improved the BSFL bin design, as shown in Figure 3. The problems with the initial BSFL bin design were the larvae escaping through the bin cover and the leachate drainage opening, which allowed house flies to enter the BSFL bin and lay eggs in the bin cover and drainage opening, and the prepupae’s reluctance to go up the ramp. These problems were rectified by installing an anti-escape system on the BSFL bin, changing the bottom-mounted leachate drainage system to side-mounted, removing the additional wooden frame intended for easy access, and using the existing lid as the access for adding food waste. We changed the aluminum ramp to plastic because aluminum absorbs heat from the surroundings, making it too hot for the prepupae to go up the ramp.

### 2.2. Medium Preparation

The food waste was pre-treated before use by removing inorganic materials, such as plastic, paper, and tissues, to prevent them from disrupting the composting process. The organic waste was pulverized and mixed well to obtain homogeneous food waste for all BSFL bins. For MCCM, the garden waste was pulverized before mixing it with the food waste, while rice bran and chicken feed were added to the food waste without pre-treatment. The food waste gathered from the UKM compost center consisted primarily of food waste with high carbohydrate contents, such as rice and noodles, high-protein food, such as chicken and fish meat, and a small portion of vegetables and fruits. Meanwhile, the garden waste was also from the UKM compost center; we then sorted the branches and collected the dry leaves to be used in this experiment.

### 2.3. Hatching the BSF Eggs

The eggs of the BSF were harvested from the BSF cage at the Faculty of Science and Technology (FST), UKM, and transferred into a small container. The small container was placed in a large plastic container containing wet chicken feed as the control feed. After rearing the hatchlings on chicken feed for eight days, we transferred about 1440 larvae to the fabricated BSFL bins set up near the Coastal Engineering Laboratory (2.9289° N, 101.7800° E) at the Faculty of Engineering and Built Environment (FKAB) to protect them from sun and rain.

### 2.4. Food Waste Treatment

**Figure 4:**
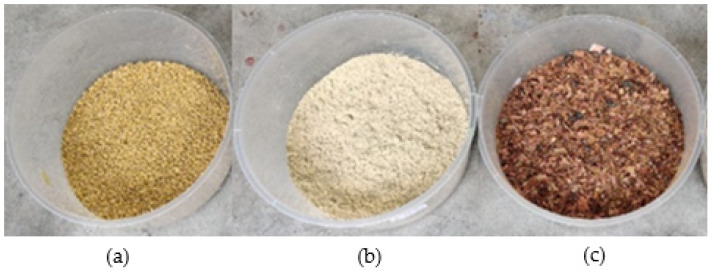
Condition of MCCM before mixing with food waste: (**a**) chicken feed; (**b**) rice bran; and (**c**) pulverized garden waste.

Table 1 shows the composition of the pulverized food waste mixed with the MCCM (chicken feed, rice bran, and garden waste), and Figure 4 shows the condition of MCCM before it was mixed with food waste. The food waste and MCCM were mixed thoroughly to obtain a homogeneous mixture and stored in different air-tight containers for each type of medium. In total, 1 kg of feeding medium was added to each BSFL bin every third day. We added the same amount of water to each mixture if they appeared too dry.

### 2.5. Laboratory Experiment

The temperature, pH, and moisture content of the fresh food waste mixtures were measured on the first day of the experiment. On every third day, we measured the humidity and temperature at the experiment site and the medium parameters, the pH, temperature, and moisture content, before adding the fresh food waste mixture to the BSFL bins. Temperature of mediums was measured by taking three random points in the bins to obtain the average readings. A sample was also taken by selecting three random points from each bin before proceeding with the analysis. For pH analysis, waste was mixed 1:1 with de-ionized water. The mixture was stirred and left to stand for 15 min before measuring the pH using a pH meter in the liquid phase. Meanwhile, moisture content was determined using a standard oven-dried technique at a temperature of 105 °C.

The amount of food waste added into each BSFL bin and the amount of frass on the final day of the experiment, day 11, were recorded to calculate the waste reduction index (WRI). We measured the weight and length of 20 larvae from each BSFL bin and calculated the average larval length and weight for each BSFL bin. On the final day of the experiment, we took 20 prepupae from the harvesting container of the BSFL bins to determine their average weight and length. We also observed the changes in the larvae, prepupae, medium, and BSFL bins.

### 2.6. Data Analysis

The ambient temperature and humidity of the experiment site were recorded for 5 days before starting the experiment. The ambient temperature ranged between 30.6 and 32.4 °C and was close to the optimum temperature of 30 °C [19]. Meanwhile, the ambient humidity was over 65%. It was concluded that the surroundings of UKM were close to optimum condition and suitable for BSF rearing. In addition, we tabulated and then plotted the graphs for the mediums’ pH, temperature, and moisture content and compared the different food wastes and MCCM compositions. The efficiency of the MCCM in reducing waste was determined by calculating the waste reduction index (WRI) for each BSFL bin on the final day of the experiment. A higher WRI value indicates better waste reduction efficiency [20].

(1) $$WRI=[\frac{\frac{W-R}{W}}{t}]\times 100\%$$

where *W* is the total quantity of feeding substrate used during the time *t*, and *R* is the residue left at harvesting time *t*.

We tabulated the average weight and length of larvae to determine the change in the larval size and compared the length and weight of the larvae reared on different food wastes and MCCM compositions. The formula below was used to calculate the growth rate (GR):

(2) $$GR=\frac{\frac{W_{1}-W_{0}}{W_{0}}\times 100\%}{t}$$

where W_1_ is the final weight of larvae at 11 days, *t*, while W_0_ is the initial weight of larvae. We conducted the same analysis on the prepupae harvested on the final day of the experiment. We also compared the changes in the medium, larvae, and prepupae from the different BSFL bins.

## 3. Results and Discussion

### 3.1. Medium

**Figure 5:**
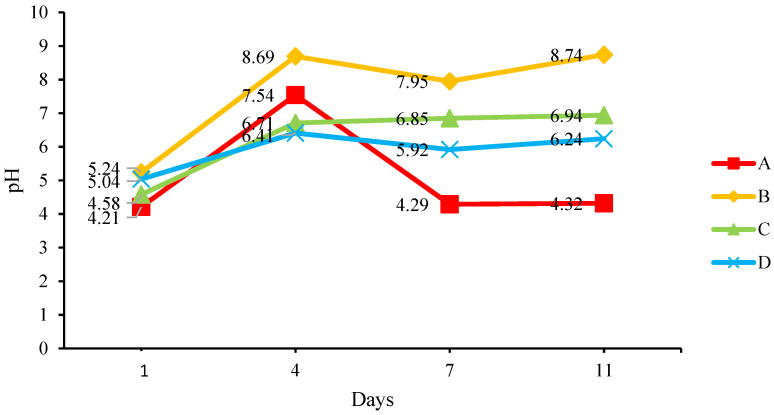
Change in the pH of the medium.

The medium for each bin was sampled to measure pH, temperature, and moisture content. Figure 5 shows the change in the measured pH of the medium. The pH value for Day 1 is for the fresh food waste mixtures, while the values for Days 4 to 11 are the pH of the frass. As anticipated, the pH of the frass in BSFL bins B, C, and D seems to increase from acidic to approaching neutral from Day 4 onwards. An increase in pH values from 5 to 8 proved that the compost was undergoing a maturing phase [21,22]. The frass in BSFL bin A showed a decreasing pH and turned acidic on Days 7 and 11 because a large percentage of the larvae were reaching the instar 4 stage faster than the larvae in other BSFL bins and had stopped consuming the food waste. Figure 5 show the sudden increase in the weight and length of the larvae in BSFL bin A. To utilize frass as a fertilizer, the optimum range is 6–8 as the bacteria population is dominant at around neutral pH, enhancing the breakdown of organic matter and cycling of nutrients for plants. Meanwhile, the rate of mineralization of nutrients by soil microbes into forms that are available to plants is slower in acidic soil, thereby restricting plant uptake. As a result, it is not ideal to utilize acidic soil as a plant fertilizer [22].

**Figure 6:**
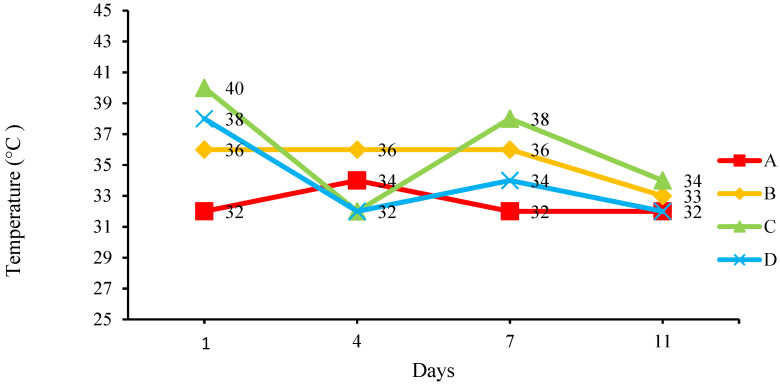
Changes in the temperature of the medium.

Figure 6 shows the change in the temperature of the medium. All BSFL bins recorded that the temperature was slightly above the ambient temperature and ranged between 32 and 40 °C. Temperatures that tend to be higher than the ambient temperature indicate that the medium is undergoing biodegradation [11]. A study has shown that the medium temperature could rise to as high as 43 °C while being decomposed by maggots, where the temperature increase helps to speed up the composting process to reach the thermophilic phase [23]. High medium temperatures exceeding 45 °C could reduce the pathogen load in the compost [24].

**Figure 7:**
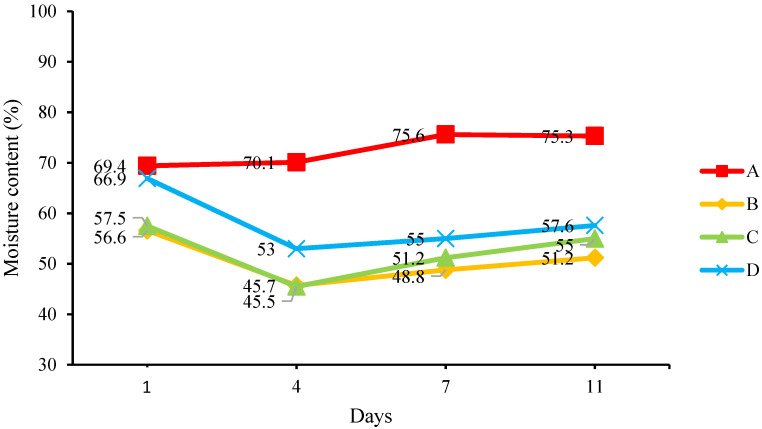
Change in the moisture content of the medium.

The change in the moisture content of the mediums from Day 1 to 11 is shown in Figure 7. The mediums with added MCCM seem to have a lower moisture content than the medium without MCCM (medium A). The difference was immediately apparent, where mixing pulverized food waste with MCCM resulted in mediums B, C, and D tending to have a slightly lower moisture content on Day 1. On Day 4, the mediums with added MCCM showed a decreased moisture content, while the moisture content of medium A increased. All mediums tend to show a similar moisture content trend on the final day of the experiment, and the moisture content of all mediums was within the optimum range. The mediums with added MCCM maintained a moisture content of 51.2 to 57.6%, while medium A without MCCM had a moisture content of over 70%. The high moisture content of medium A caused the frass to be moist, sticky, and foul-smelling. Dortmans [24] observed that a moisture content exceeding 60% caused nutrient leachate and foul odor, similar to BSFL bin A, while a moisture content of less than 40% hindered the growth of larvae and bacteria [25]. The moisture content data showed that food waste mixed with MCCMs produced lower moisture content of frass at final day compared to food waste without MCCMs. Although the growth rate of BSFL increased with higher moisture contents (BSFL can survive in medium with a moisture content as high as 80%), the high moisture contents caused problems in maintenance and waste separation work [26]. These findings indicate a trade-off between BSFL growth, ease of maintenance, and leachate prevention.

**Figure 8:**
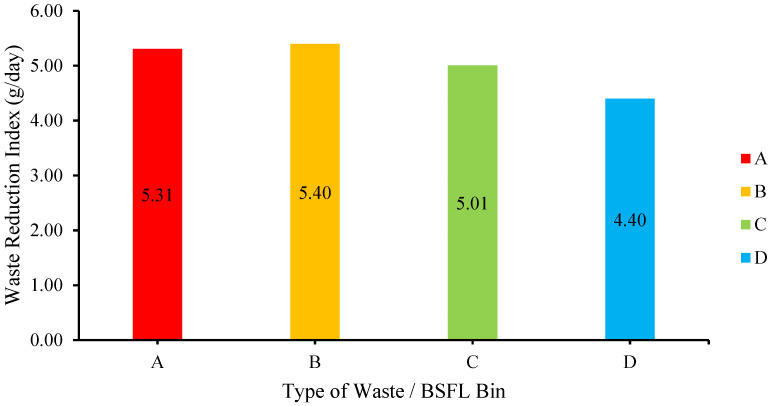
Waste Reduction Index (WRI).

Figure 8 shows the WRI for each medium. It shows that medium B seems to have the highest WRI of 5.40 g/day, followed by medium A (5.31 g/day). As anticipated, medium D had the lowest WRI of 4.40 g/day since BSFL cannot decompose waste with high cellulose contents; in this case, the garden waste added to the medium was virtually untouched [27]. This study hypothesized that medium A should have the highest WRI. During the experiment, excessive food waste was added from Day 7 onwards, even though most larvae had reached the prepupae stage and stopped eating. The large portion of uneaten food waste resulted in a lower WRI. Nonetheless, the WRI showed that medium B containing chicken feed produced the best result.

### 3.2. Larvae and Prepupae

**Figure 9:**
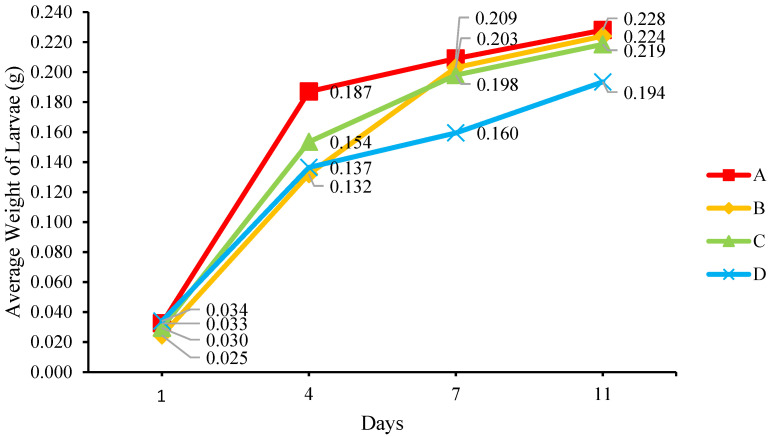
The average weight of the larvae (g).

**Figure 10:**
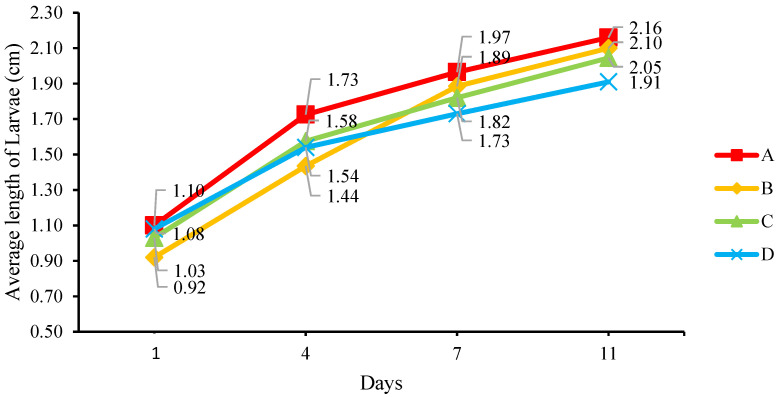
The average length of the larvae (cm).

Figure 9 and Figure 10 show the average weight and length of the larvae, while Table 2 shows the larval growth rate in terms of weight and length. The larvae reared on all mediums tend to have a similar weight and length pattern, where their growth surged on Day 4 and decreased from Day 7 to Day 11. The larvae reared on medium A reached the peak growth rate quicker than those on other mediums, and a large percentage of the larvae reached the instar 4 stage on Day 4, after which they grew at a slower rate. On Days 7 and 11, the growth rate of the larvae reared on mediums B and C exceeded those on medium A, but they were slightly smaller. The highest growth rate in weight (72.36%) and length (11.66%) was recorded in the larvae reared on medium B on the final day of the experiment. However, the average weight and length of the larvae reared on medium B were lower than those on medium A, where the final weight and length of the larvae on medium B were 0.224 g and 2.10 cm, and those on medium A weighed 0.228 g and were 2.16 cm long. The larval sizes in this experiment were similar to those recorded by other researchers [26,28], where the highest weight was 0.200–0.225 g. The larvae reared on medium A had the best average weight and length, followed by the larvae on medium B; there is a possibility that the size of the larvae reared on medium B would exceed those reared on medium A if the duration of the experiment was longer. Despite this, medium A produced a higher moisture content of frass compared to medium B, based on Figure 9, which seems to show that MCCM is necessary in controlling moisture content and enhancing the quality of frass. A comparison of the medium with added MCCM showed that medium B containing chicken feed had the best overall result, indicating that chicken feed is the best MCCM for obtaining the heaviest and longest larvae. It has been proven by previous studies that the higher protein content of larval feeding medium produced better larval growth [22,29].

Table 3 shows the average weight and length of the prepupae from the harvesting container. The average weight and length of the prepupae are similar to those of the larvae on Day 11. The prepupae from BSFL bin A tend to have the highest weight (0.225 g) and length (2.15 cm), followed by the larvae from BSFL bin B (0.221 g and 2.11 cm). As anticipated, the larvae from BSFL bin D seem to have the lowest weight and length of 0.195 g and 1.89 cm. Previous studies have shown that larvae reared on vegetables had the lowest growth rate because vegetables have a high fiber content and are difficult to digest [30,31]. The average weight of the prepupae in this experiment was lower than the average weight of the larvae on Day 11, which is similar to the result obtained by Cheng et al. [26] and Kim et al. [32], because the prepupae ceased feeding and began to eliminate the remaining food from their digestive systems in preparation to pupate. In this stage, the prepupae use the reserve fats in their body to generate energy since movements and metamorphosis require energy, as reported by Mirwandhono et al. [33].

The analysis of crude protein and crude fat results showed that the use of different MCCM had no significant effect. Table 4 shows that the larvae reared on food waste in bin A (47.95%) seem to have higher protein composition than those feeding in another bin. The larva reared in bin B, which contained a mixture of food waste and chicken feed, have the highest larval fat content (38.6%). According to Wang et al. [34], food waste has a higher protein content than pig manure, cow dung, and chicken manure. Numerous investigations have assessed the efficacy of BSF meal as a poultry feed [35]. The results generally indicate that BSF meal can be a viable replacement for a significant portion of soybean meal in poultry diets without affecting product performance or quality [36]. Additionally, studies have successfully used BSF meal as a substitute for fish meal in several fish species, such as channel catfish (*Ictalurus punctatus*) [37], African catfish (*Clarias gariepinus*) [38], Nile tilapia (*Oreochromis niloticus*) [39], rainbow trout (*Oncorhynchus mykiss*) [39], and Atlantic salmon (*Salmo salar*) [40]. These findings suggest that BSF meal has the potential to serve as an alternative protein source for various fish species.

### 3.3. Observation

**Figure 11:**
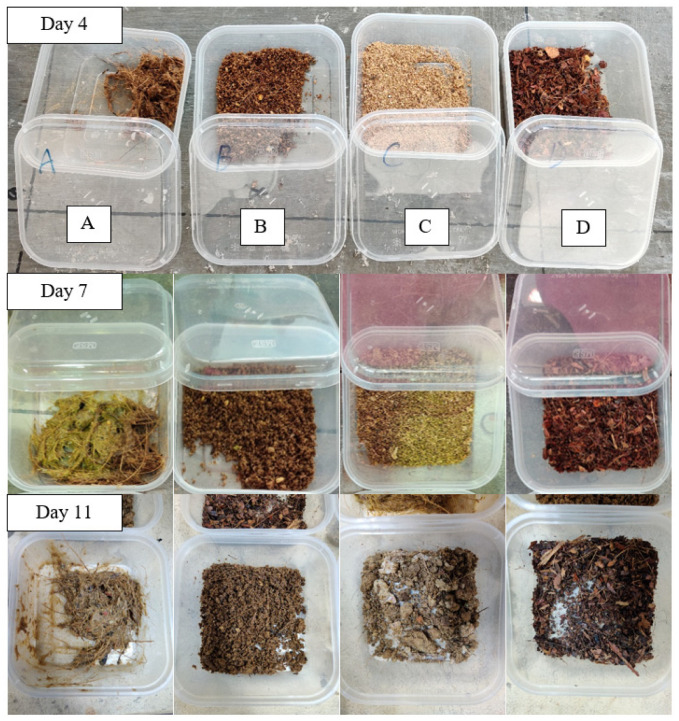
The physical changes in the frass.

We recorded the physical changes in the frass, larvae, collected prepupae, and other parameters, such as the condition in each BSFL bin and the eggies. Observation of the frass in the BSFL bins revealed a difference between the mediums with and without MCCM. The frass from BSFL bins B, C, and D was much drier than that from BSFL bin A as show in Figure 11. The frass in BSFL bin A was watery, sticky, and foul-smelling; its condition gradually worsened as the experiment progressed, and a small amount of stagnant leachate accumulated at the base of the BSFL bin. The liquid from the frass helped the larvae climb the wall of BSFL bin A and damaged the anti-escape system in the BSFL bin on Day 7. The frass also made it difficult to manage the composting system, including cleaning, maintaining, and separating the larvae from the frass. The frass from BSFL bins B, C, and D was dry and easy to separate from the larvae, and the moisture content increased from Day 7 onwards. The leftover food waste in BSFL bins B and C damaged some of the frass, making it lumpy on Day 7. Only the frass from BSFL bin D remained dry and fine until the final day of the experiment since the larvae consumed all food waste. In summary, the garden waste in BSFL bin D helped produce the best frass.

The physical changes in the larvae revealed that the size of the larvae in all BSFL bins increased by at least three-fold on Day 4. A comparison of the size of the larvae from each BSFL bin showed that those reared on medium A were the largest, while the larvae reared on medium D were smaller. The observations remained unchanged until the last day of the experiment except for the minor differences in the size of the larvae fed with different mediums. On Day 7, more larvae from BSFL bins A and B reached the prepupae stage compared to those from BSFL bins C and D.

**Figure 12:**
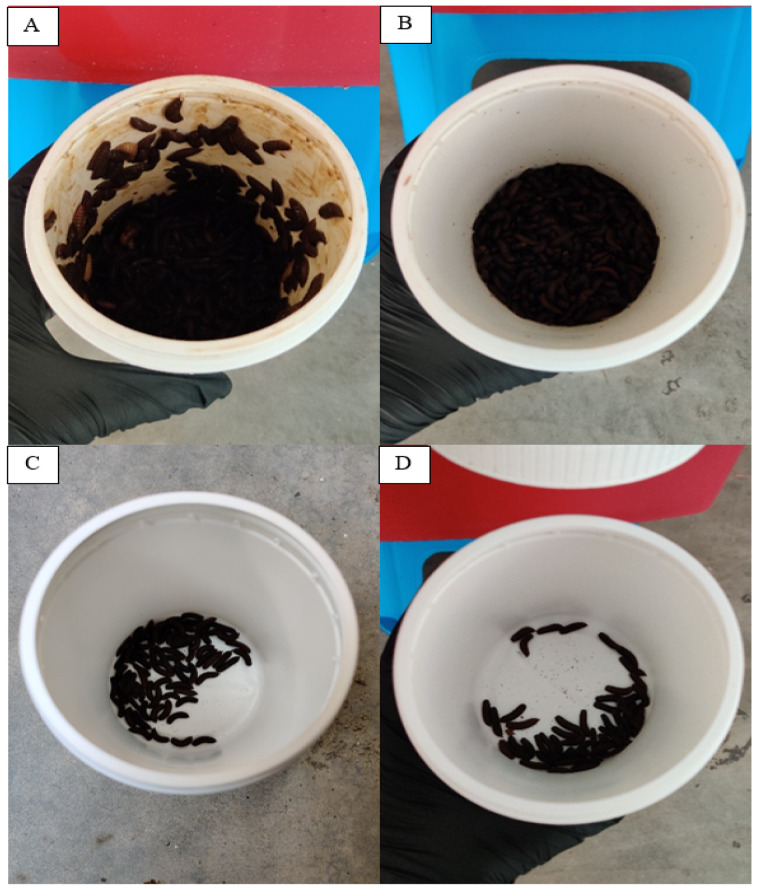
The harvesting container on second rearing cycle Day 11.

Concerning the effectiveness of the self-harvesting system, the harvesting containers in BSFL bins A and B were almost 1/3 filled on the last day of the experiment as shown in Figure 12. However, the harvesting containers in BSFL bins C and D only had a few prepupae. Even though the harvesting container of BSFL bins A and B have about the same number of prepupae, the watery frass in BSFL bin A caused the prepupae to climb the container wall, some of which escaped when we opened the container, and made it difficult to handle the prepupae. There were several larvae in the instar 4 or 5 phase in the harvesting container. The three BSFL bins containing MCCM were easy to handle since there were no escaping prepupae. Most prepupae in BSFL bins C and D did not migrate to the harvesting container and were resting in the BSFL bins. BSFL bin B was the most effective self-harvesting system, and BSFL bins A, C, and D were the least effective.

## 4. Conclusions

In conclusion, this study has successfully designed and tested a user-friendly BSFL bin for treating organic waste. The results have shown that the fabricated BSFL bins can fulfill the BSF lifecycle requirements, with wild BSFs laying eggs in mediums such as food waste and then the hatched larvae decomposing it. The by-products from this system are prepupae, which can be directly fed to farm animals such as chickens [41], and frass, which can be used as a plant fertilizer [15]. This system seems to be theoretically a continuous system, as this composting bin is recommended to be placed at open sites near vegetative areas [20] to attract wild BSFs to lay eggs in the bin and sustain the larvae. The addition of MCCMs, such as chicken feed, rice bran, and garden waste, to the food waste has shown to be effective in reducing the moisture content and producing larvae and prepupae with optimal growth rates. The use of chicken feed as the MCCM resulted in the highest growth rate and frass with the lowest moisture content. The findings of this study have important implications for addressing the challenges associated with organic waste generation and landfill sites. Future research can explore the potential of using different types of MCCMs and waste materials to optimize the growth of larvae and prepupae, as well as investigate the potential of using the harvested prepupae and frass as sources of protein and fertilizer, respectively. Overall, the use of BSFL composting systems has great potential as a sustainable and cost-effective method for treating organic waste.

## Figures and Tables

**Table 1:** Composition of food waste and MCCM.

BSFL Bin	MCCM	Composition (Weight)(%)	
		Food Waste	MCCM
A	Control	100%	-
B	Chicken feed	75%	25%
C	Rice bran	75%	25%
D	Garden waste	75%	25%

**Table 2:** Larval growth rate in terms of weight and length.

Type of Waste	Growth Rate (%)	
	Weight	Length
A	53.72	8.76
B	72.36	11.66
C	57.27	9.00
D	42.78	6.99

**Table 3:** Average weight and length of prepupae.

Type of Waste	Average	
	Weight (g)	Length (cm)
A	0.225	2.15
B	0.221	2.11
C	0.219	2.07
D	0.195	1.89

**Table 4:** Crude protein and crude fat analysis of black soldier fly maggots using MCCM media.

BSFL Bin	MCCM	Crude Protein (%)	Fat (%)
A	100% Food Waste (Control)	47.95	29.2
B	75% Food Waste + 25% Chicken feed	45.6	38.6
C	75% Food Waste + 25% Rice bran	42.8	36.5
D	75% Food Waste + 25% Garden waste	37.8	30.8

## Data Availability

Data are contained within this article.
